# The nature of inter- and intramolecular interactions in F_2_OXe^…^HX (X= F, Cl, Br, I) complexes

**DOI:** 10.1007/s00894-016-2970-8

**Published:** 2016-05-04

**Authors:** Emilia Makarewicz, Jan Lundell, Agnieszka J. Gordon, Slawomir Berski

**Affiliations:** Faculty of Chemistry, University of Wroclaw, F. Joliot-Curie 15, 50-383 Wroclaw, Poland; Department of Chemistry, University of Jyväskylä, PO Box 35, 40014 Jyväskylä, Finland

**Keywords:** ELF, Quantum chemical topology, SAPT, Noble gas complexes, Xenon

## Abstract

Electronic structure of the XeOF_2_ molecule and its two complexes with HX (X= F, Cl, Br, I) molecules have been studied in the gas phase using quantum chemical topology methods: topological analysis of electron localization function (ELF), electron density, ρ(r), reduced gradient of electron density |RDG(r)| in real space, and symmetry adapted perturbation theory (SAPT) in the Hilbert space. The wave function has been approximated by the MP2 and DFT methods, using APF-D, B3LYP, M062X, and B2PLYP functionals, with the dispersion correction as proposed by Grimme (GD3). For the Xe-F and Xe=O bonds in the isolated XeOF_2_ molecule, the bonding ELF-localization basins have not been observed. According to the ELF results, these interactions are not of covalent nature with shared electron density. There are two stable F_2_OXe^…^HF complexes. The first one is stabilized by the F-H^…^F and Xe^…^F interactions (type I) and the second by the F-H^…^O hydrogen bond (type II). The SAPT analysis confirms the electrostatic term, E_elst_^(1)^ and the induction energy, E_ind_^(2)^ to be the major contributors to stabilizing both types of complexes.

## Introduction

**Scheme 1:**
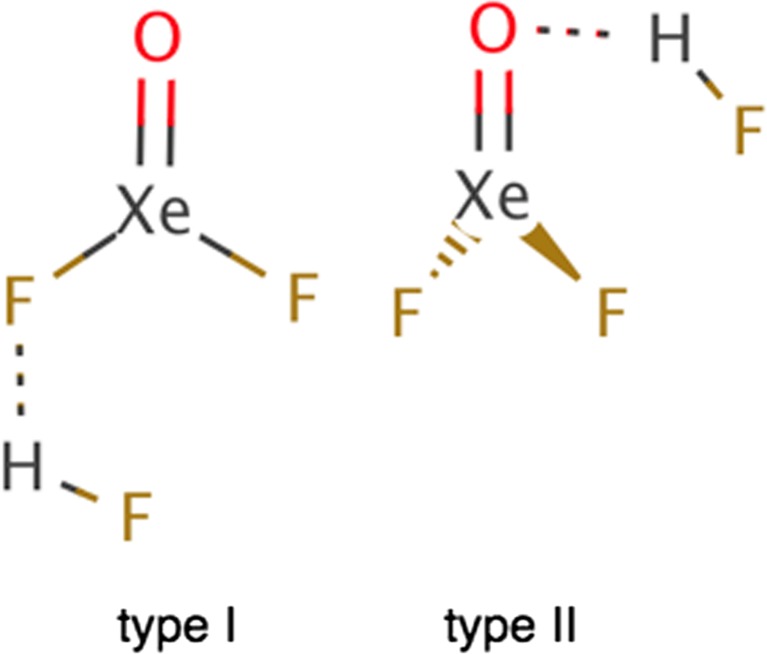
The F_2_OXe^…^HF complexes identified experimentally by Brock et al. [3]

The XeOF_2_ molecule, with the xenon atom formally in oxidation state +4, was first observed by Ogden and Turner [1] in 1966 and subsequently by Jacob and Opferkuch [2] in 1976. Intermolecular complexes of XeOF_2_ with hydrogen fluoride (F_2_OXe^…^HF) have been synthesized and characterized by the Schrobilgen group [3], using vibrational spectroscopy and computational methods (Scheme 1). The most interesting result stemming from those experimental studies is stabilization of the F_2_OXe^…^HX complex with the weak F-H^…^O and F-H^…^F hydrogen bonds and weak Xe^…^F interactions. A detailed nature of the xenon–fluorine interaction is currently not entirely understood and the state-of-art electronic structure analysis is crucial to gain a deeper insight into this interaction.

Topological analysis of electron density field, ρ(r) proposed by Bader [4] and known as atoms in molecules theory (AIM), topographical analysis of localized electron detector (LED) [5, 6] or the non-covalent index (NCI) [7], both based on the magnitude of the reduced gradient of electron density (|RDG(r)|), can fully characterize all bonding and non-bonding interactions, without a need to evoke the molecular orbital concept. On the other hand, topological analysis of electron localization function, η(r), (ELF) [8, 9], serves best as a tool for covalent bonding analysis.

The current paper presents optimized geometrical structures of the F_2_OXe^…^HX (X= F, Cl, Br, I) complexes in the gas phase together with theoretical properties of intermolecular interactions. Non-covalent intermolecular interactions are described using topological analysis of electron density, ρ(r) and |RDG(r)|. Detailed analysis of the electronic structure of the isolated XeOF_2_ molecule and its intermolecular complexes with hydrogen fluoride, HF, has been performed using topological analysis of ρ(r), and η(r) fields. Finally, the nature of non-covalent intermolecular interactions in the F_2_OXe^…^HF has been examined using the symmetry adapted perturbation theory (SAPT) [10].

## Computational details

Full optimization of geometrical structures together with calculated vibrational spectra have been carried out using the Gaussian09 programme [11]. The wave function has been approximated by the MP2 [12, 13] and DFT calculations using APF-D [14], B3LYP [15], M062X [16], and B2PLYP [17] functionals, augmented with the Grimme dispersion correction (GD3) [18]. The CCSD(T) calculations have been performed using the MOLPRO program [19].

The APF-D functional, based on the new hybrid density functional, APF, includes the empirical dispersion model (D) [14]. The functional uses a spherical atom model for the instantaneous dipole–induced dipole interactions. The functional correctly describes a large portion of the potential energy surface (PES) for noble gas complexes with various diatomic molecules [14]. The B2PLYP functional [20] combines the exact HF exchange with an MP2-like correlation in the DFT calculation, and belongs to the final fifth rung of the Jacob’s ladder, introduced by Perdew [21]. It incorporates information about the unoccupied Kohn–Sham orbitals.

In the Def2-TZVPPD basis set [22] 28 electrons been replaced by the pseudopotential (ecp-28) for both Xe and I atoms. The minima on the potential energy surface (PES) have been confirmed through non-imaginary frequencies in the harmonic vibrational analysis.

Interaction energies, defined as a difference between the total energy of the complex and its monomers with geometrical structures corresponding to the complex (E_int_), have been corrected using basis set superposition error (BSSE) (E_int_^CP^) obtained with the counterpoise procedure proposed by Boys and Bernardi [23]. The differences between the E_tot_ values for the complex and optimized geometrical structures (equilibrium geometry) for the isolated monomers, dissociation energy ΔE_dis,_ have been corrected for the vibrational zero-point energy correction (ΔE_dis_ + ΔZPVE). The final E_int_^CP^ value also includes the vibrational zero-point energy correction, (E_int_^CP^ + ΔZPVE).

Topological analysis of electron density, ρ(r), has been carried out using the AIMAll program [24] with the DFT(M062X) wave function, calculated for the geometrical structures, optimized at the DFT(M062X)/Def2-TZVPPD computational level. The wfx files containing additional information for the atomic region, described by ecp-28, have been used.

Reduced gradients of the electron density have been calculated using the AIMAll program with the wave function approximated at the DFT(B3LYP)/TZP//DFT(M062X)/Def2-TZVPPD level.

Topological analysis of ELF has been performed using the TopMod09 package [25] with the wave function approximated using the DFT(B3LYP)/Def2-TZVPPD single-point calculations for geometrical structures optimized at the DFT(M062X)/Def2-TZVPPD computational level. The parallelepipedic grid of points with step 0.05 bohr has been used.

SAPT analysis has been performed using the MOLPRO (Version 2012.1) program [19] for the geometrical structures optimized at the B2PLYP + GD3/Def2-TZVPPD computational level.

The Def2-TZVPPD and TZP [26–28] basis sets have been obtained from the EMSL Basis Set Library using the Basis Set Exchange software [29, 30].

## Results and discussion

### Geometrical structure and interaction energy

**Fig. 1:**
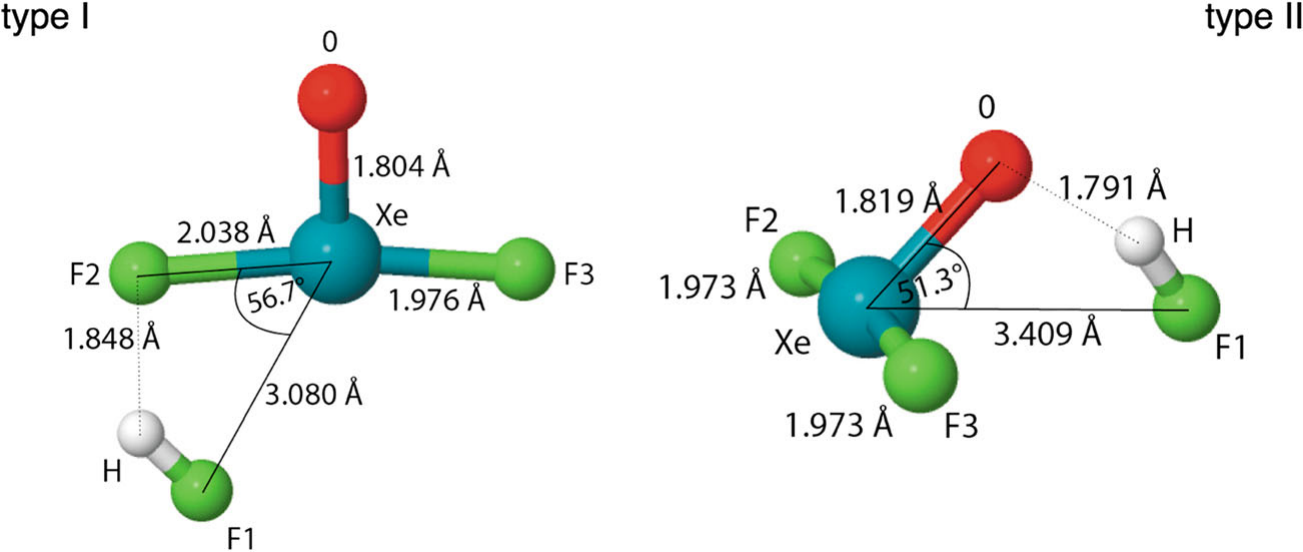
Geometrical structures of two types of the F_2_OXe^…^HF complexes, optimized at the DFT(B3LYP)/Def2-TZVPPD level – type I, stabilized by the F-H^…^F and Xe^…^F interactions and type II, stabilized by the F-H^…^O interaction

Geometrical structures of the intermolecular F_2_OXe^…^HX (X= F, Cl, Br, I) complexes have been optimized using a variety of density functionals and the MP2 method. Optimized geometrical structures are shown in Fig. 1. For the F_2_OXe^…^HX (X= F, Cl, Br, I) complexes, two minima on the PES have been found. Structural differences between complexes (type I and type II) lie mainly in the orientation of the HX molecule with respect to the XeOF_2_ molecule. The optimized geometrical parameters for all the F_2_OXe^…^HX complexes are shown in Table 1 (type I) and Table 2 (type II). The parameters obtained with the DFT(M062X + GD3) method have been omitted since the addition of the dispersion correction did not bring any changes. Only complexes with the HF molecule are discussed and compared to the existing experimental results [3]. Optimizations performed at the highest computational level, CCSD(T)/Def2-TZVPPD, yield the following results: Xe^…^F of 3.015 Å, F^…^H 1.860 Å, F-H 0.930 Å, and F^…^F 2.856 Å for the type I and the O^…^H of 1.790 Å, F-H 0.934 Å and F^…^O 2.663 Å for the type II. It is worth noting, that the Xe^…^F distance is best reproduced by the B3LYP functional (3.022 Å) when compared to the results at the CCSD(T) level.

**Table 1:** The optimized geometrical parameters for the structure type I of the F_2_OXe^…^HX (X= F, Cl, Br, I) complexes

Param/method:	MP2	APFD	M062X	B3LYP	B3LYP + GD3	B2PLYP	B2PLYP + GD3
HF							
Xe-O	1.763	1.785	1.789	1.804	1.804	1.795	1.795
Xe^…^F	2.938	2.915	2.929	3.022	3.080	2.980	3.006
H^…^F2	1.844	1.801	1.894	1.813	1.848	1.824	1.850
F2^…^X^…^F1	59	59	59	57	57	58	58
HCl							
Xe-O	1.765	1.788	1.790	1.806	1.806	1.797	1.797
Xe^…^Cl	3.368	3.359	3.422	3.5	3.543	3.448	3.482
H^…^F2	2.207	2.260	2.231	2.305	2.234	2.245	2.245
F2^…^Xe^…^Cl	66	67	64	65	64	65	64
HBr							
Xe-O	1.766	1.788	1.790	1.807	1.806	1.798	1.797
Xe^…^Br	3.451	3.439	3.559	3.628	3.690	3.557	3.591
H^…^F2	2.383	2.383	2.328	2.538	2.306	2.442	2.337
F2^…^Xe^…^Br	70	70	66	69	66	69	67
HI							
Xe-O	1.767	1.790	1.791	1.809	1.808	1.799	1.798
Xe^…^I	3.593	3.588	3.729	3.784	3.847	3.703	3.758
H^…^F2	2.732	2.809	2.542	3.658	2.658	3.003	2.605
F2^…^Xe^…^I	75	76	69	85	71	76	71

**Table 2:** The optimized geometrical parameters for the structure type II of the F_2_OXe^…^HX (X= F, Cl, Br, I) complexes

Param/method:	MP2	APFD	M062X	B3LYP	B3LYP + GD3	B2PLYP	B2PLYP + GD3
HF							
Xe-O	1.777	1.800	1.803	1.819	1.819	1.809	1.808
Xe^…^F	3.273	3.205	3.128	3.424	3.409	3.354	3.359
O^…^H	1.782	1.746	1.824	1.779	1.791	1.790	1.799
O-Xe^…^F	54	53	58	51	51	53	53
HCl							
Xe-O	1.773	1.797	1.798	1.806	1.814	1.804	1.804
Xe^…^Cl	3.664	3.627	3.577	3.986	3.828	3.852	3.785
O^…^H	1.944	1.893	2.052	2.305	2.006	1.994	2.000
O-Xe^…^Cl	59	59	63	53	57	57	58
HBr							
Xe-O	1.773	1.797	1.797	1.814	1.813	1.803	1.813
Xe^…^Br	3.771	3.707	3.750	4.174	3.967	4.011	3.967
O^…^H	1.955	1.904	2.055	2.069	2.050	2.032	2.050
O-Xe^…^Br	60	61	63	54	58	57	59
HI							
Xe-O	1.770	1.795	1.796	1.796	1.812	1.811	1.802
Xe^…^I	3.941	3.906	3.904	3.905	4.542	4.181	4.231
O^…^H	2.058	2.012	2.236	2.238	2.235	2.183	2.177
O-Xe^…^I	64	64	67	67	55	62	60

**Fig. 2:**
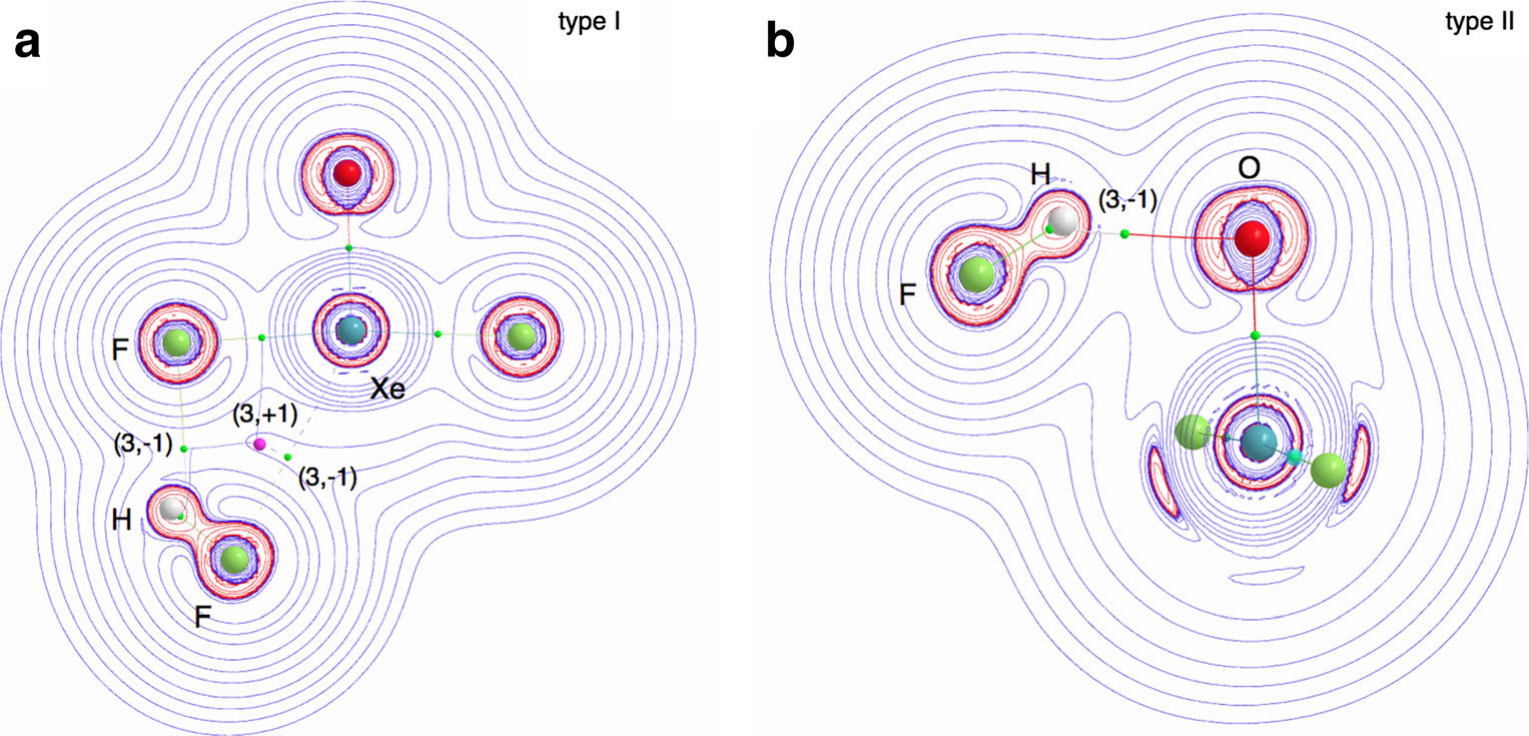
The critical points of the ρ(r) field and 2D maps of the Laplacian of ρ(r) field for the F_2_OXe^…^HF complexes

The type I complex is stabilized by the F-H^…^F hydrogen bond and the Xe^…^F non-bonding interaction, confirmed by bond paths with the bond critical points (BCP) localized for the gradient field of ρ(r) (see Fig. 2a). The F-H^…^F hydrogen bond is topologically characterized by relatively large electron density for the BCP (ρ_BCP_(r) = 0.025 e/bohr^3^) and positive value of the Laplacian electron density for the BCP (∇^2^ρ_BCP_(r) = 0.122 e/bohr^5^) (see Table 3). Supposedly weaker Xe^…^F interaction is characterized by smaller ρ_BCP_(r) (0.016 e/bohr^3^) and smaller and positive ∇^2^ρ_BCP_(r) (0.070 e/bohr^5^). The (3,-1) CP between Xe and F nuclear attractors is localized in a proximity of the (3,+1) CP. The type II complex is stabilized only by the F-H^…^O hydrogen bond (ρ_BCP_(r) = 0.033 e/bohr^3^, ∇^2^ρ_BCP_(r) = 0.107 e/bohr^5^) and the BCP characterizing this interaction is shown in Fig. 2b. The ρ_BCP_(r) value is larger than that obtained for the F-H^…^F hydrogen bond in the type I. The difference can be caused by stronger intermolecular interaction.

**Table 3:** Properties of the bond critical point (BCP) and delocalization index values for the type I and type II F_2_OXe^…^HF complexes. All values are in atomic units

A–B	δ(A,B)	ρ_BCP_	∇^2^ρ_BCP_(r)	H_BCP_	ε_BCP_
type I					
	intermolecular interactions				
H^…^F	0.035	0.025	0.122	0.003	0.310
Xe^…^F	0.087	0.016	0.070	0.003	0.227
	intramolecular interactions				
F-H	0.376	0.354	−3.014	−0.835	0.001
Xe-O	1.539	0.213	0.201	−0.157	0.030
Xe-F	0.819	0.141	0.271	−0.078	0.114
Xe-F_(F-H…F)_	0.731	0.126	0.236	−0.064	0.129
type II					
	intermolecular interactions				
H^…^O	0.059	0.033	0.107	−0.003	0.058
	intramolecular interactions				
F-H	0.361	0.348	−2.910	0.813	0.002
Xe-O	1.459	0.208	0.170	−0.151	0.055
Xe-F	0.817	0.142	0.271	−0.079	0.114
Xe-F	0.817	0.141	0.270	−0.079	0.115

*δ(A,B)* delocalization index for pair of A,B atoms, *ρ*_*BCP*_ electron density for BCP, *∇* ^*2*^ *ρ*_*BCP*_ *(r)* Laplacian of electron density for BCP, *H*_*BCP*_ total energy density for BCP, *ε*_*BCP*_ ellipticity for BCP

The strength of intermolecular interaction has been evaluated using supermolecular approach with two parameters: the interaction energy, (E_int_^CP^, E_int_^CP^ + ΔZPVE) and the dissociation energy (ΔE_dis_ + ΔZPVE). Values for the F_2_OXe^…^HX (X= F, Cl) complexes have been presented in Table 4, and for the F_2_OXe^…^HX (X=Br, I) complexes in Table 5. During discussion we will concentrate on the values of E_int_^CP^ + ΔZPVE only.

**Table 4:** Values of the interaction (E_int_) and dissociation (ΔE_dis_) energies corrected for the basis superposition error (BSSE) and zero-point vibrational energies (ΔZPVE) for both geometrical structure types of the F_2_OXe^…^HX (X= F, Cl) complexes. All energies are given in kcal/mol

Molecule:	HF										HCl									
Structure:	Type I					Type II					Type I					Type II				
Method/param:	E_int_	E^CP^_int_	E^CP^_int_ + ΔZPVE	ΔE_dis_	ΔE_dis_ + ΔZPVE	E_int_	E^CP^_int_	E^CP^_int_ + ΔZPVE	ΔE_dis_	ΔE_dis_ + ΔZPVE	E_int_	E^CP^_int_	E^CP^_int_ + ΔZPVE	ΔE_dis_	ΔE_dis_ + ΔZPVE	E_int_	E^CP^_int_	E^CP^_int_ + ΔZPVE	ΔE_dis_	ΔE_dis_ + ΔZPVE
MP2	−7.82	−6.62	−4.97	−7.16	−5.51	−7.36	−6.15	−4.29	−6.97	−5.11	−5.54	−4.66	−5.66	−5.34	−4.35	−5.58	−4.43	−5.45	−5.36	−4.35
APFD	−8.12	−7.87	−6.21	−7.15	−5.49	−7.91	−7.76	−5.80	−7.54	−5.58	−5.41	−5.27	−6.24	−5.09	−4.11	−5.59	−5.42	−6.70	−5.39	−4.11
M062X	−8.21	−7.9	−6.07	−7.44	−5.62	−7.96	−7.77	−5.85	−7.78	−5.86	−5.14	−4.93	−5.81	−4.79	−3.91	−5.11	−4.94	−6.12	−5.09	−3.91
M062X + GD3	−8.24	−7.93	−6.10	−7.48	−5.65	−7.99	−7.79	−5.87	−7.80	−5.88	−5.14	−4.93	−5.83	−4.83	−3.93	−5.14	−4.97	−6.16	−5.12	−3.93
B3LYP	−6.11	−6.01	−4.40	−5.70	−4.10	−6.11	−6.01	−4.15	−5.82	−3.96	−3.09	−2.97	−3.82	−2.86	−2.01	−3.07	−2.92	−3.90	−2.99	−2.01
B3LYP + GD3	−6.55	−6.34	−4.78	−6.82	−5.26	−7.38	−7.28	−5.41	−7.11	−5.24	−5.07	−4.93	−5.89	−4.81	−3.85	−5.11	−4.97	−6.16	−5.05	−3.85
B2PLYP	−7.2	−6.7	−6.71	−5.79	−5.80	−6.46	−6.04	−4.66	−5.69	−4.31	−4.06	−3.73	−2.81	−1.99	−2.91	−3.86	−3.45	−3.27	−2.73	−2.91
B2PLYP + GD3	−7.8	−7.32	−7.39	−5.42	−5.49	−7.37	−6.96	−5.56	−6.48	−5.08	−5.22	−4.87	−4.07	−3.20	−4.00	−5.11	−4.7	−4.63	−3.93	−4.00

**Table 5:** Values of the interaction (E_int_) and dissociation (ΔE_dis_) energies corrected for the basis superposition error (BSSE) and zero-point vibrational energies (ΔZPVE) for both geometrical structure types of the F_2_OXe^…^HX (X=Br, I) complexes. All energies are given in kcal/mol

Molecule:	HBr										HI									
Structure:	Type I					Type II					Type I					Type II				
Method/param:	E_int_	E^CP^_int_	E^CP^_int_ + ΔZPVE	ΔE_dis_	ΔE_dis_ + ΔZPVE	E_int_	E^CP^_int_	E^CP^_int_ + ΔZPVE	ΔE_dis_	ΔE_dis_ + ΔZPVE	E_int_	E^CP^_int_	E^CP^_int_ + ΔZPVE	ΔE_dis_	ΔE_dis_ + ΔZPVE	E_int_	E^CP^_int_	E^CP^_int_ + ΔZPVE	ΔE_dis_	ΔE_dis_ + ΔZPVE
MP2	−5.62	−4.45	−5.26	−5.48	−4.67	−5.56	−4.11	−5.27	−5.36	−4.20	−5.53	−4.32	−4.94	−5.44	−4.82	−4.97	−3.48	−4.43	−4.86	−3.91
APFD	−5.55	−5.39	−6.32	−5.30	−4.37	−5.43	−5.24	−6.55	−5.26	−3.96	−5.29	−5.17	−5.90	−5.09	−4.36	−4.3	−4.17	−5.25	−4.20	−3.13
M062X	−4.8	−4.61	−6.18	−4.56	−2.99	−4.65	−4.45	−6.24	−4.75	−2.96	−4.2	−4.08	−5.14	−4.01	−2.94	−3.75	−3.61	−4.84	−3.77	−2.53
M062X + GD3	−4.83	−4.63	−6.20	−4.60	−3.02	−4.68	−4.48	−6.27	−4.78	−2.99	−4.23	−4.12	−5.16	−4.04	−2.99	−3.77	−3.63	−4.81	−3.79	−2.61
B3LYP	−2.66	−2.54	−3.44	−2.53	−1.63	−2.45	−2.3	−3.47	−2.44	−1.27	−2.31	−2.25	−2.82	−2.29	−1.72	−1.41	−1.33	−2.13	−1.45	−0.65
B3LYP + GD3	−4.81	−4.68	−5.50	−4.60	−3.78	−4.78	−4.64	−5.81	−4.77	−3.60	−4.29	−4.22	−4.99	−4.18	−3.41	−3.93	−3.84	−4.84	−3.94	−2.94
B2PLYP	−3.74	−3.33	−2.12	−1.52	−2.73	−3.43	−2.94	−2.81	−2.09	−2.23	−3.4	−3	−1.23	−0.94	−2.71	−2.52	−2.08	−1.55	−1.07	−1.60
B2PLYP + GD3	−4.98	−4.57	−3.34	−2.74	−3.98	−4.86	−4.36	−4.18	−3.45	−3.63	−4.53	−4.14	−2.47	−2.10	−3.77	−4.09	−3.63	−3.02	−2.48	−3.09

The E_int_^CP^ + ΔZPVE values for all complexes (type I and II) are smaller than −7.39 kcal/mol at the DFT level (HF, B2PLYP + GD3) and smaller than −5.66 kcal/mol at the MP2 level (HCl). These results confirm weak bonding in the studied complexes. The MP2 results and most of the DFT functionals yield larger stability of the F_2_OXe^…^HF complex, supported by the F-H^…^F and Xe^…^F interactions (type I). The B3LYP + GD3 method is an exception, yielding slightly larger stability for the type II complex with a very small difference of 0.63 kcal/mol. For other functionals the difference between both forms varies between 0.22 kcal/mol (M062X) and 2.05 kcal/mol (B2PLYP). When the F atom is replaced by a less electronegative Cl, the stability order changes and all the DFT calculations show the type II to be more stable due to the existence of the Cl-H^…^O hydrogen bond. Nevertheless, the differences between the E_int_^CP^ + ΔZPVE values for both forms are very small (0.08 kcal/mol (B3LYP) and 0.56 kcal/mol (B2PLYP + GD3)). Similar results have been obtained for the F_2_OXe^…^HBr complex, with the type II complex also more stable with all the DFT functionals used. The differences between both complexes range between 0.01 kcal/mol (MP2) and 0.84 kcal/mol (B2PLYP + D3). For the XeOF_2_^…^HI complex, all three (M062X, B3LYP, APF-D) DFT functionals (also B3LYP + GD3 and M062X + GD3) and the MP2 method show the type I as more stable, due to the I-H^…^F and Xe^…^I interactions. The differences in the E_int_^CP^ + ΔZPVE vary between 0.15 kcal/mol (B3LYP + GD3) and 0.69 kcal/mol (B3LYP). Only the B2PLYP and B2PLYP + GD3 functionals yield slightly larger stability for the type II complex. The E_int_^CP^ + ΔZPVE differences are 0.32 and 0.55 kcal/mol, respectively. The differences in energy are generally smaller than 1 kcal/mol, therefore calculations at a higher computational level, CCSD(T)/Def2-TZVPPD, has been used in order to establish the relative stability of both structures. The E_int_^CP^ (E_int_^CP^ + ΔZPVE) for the type I obtained this way is −6.32 (−4.67) kcal/mol and −6.16 (−4.32) kcal/mol for the type II, thus the complex stabilized with the F-H^…^F hydrogen bond and Xe^…^F interaction is slightly more stable (0.16 kcal/mol - ΔE_int_^CP^). The CCSD(T) level yield similarly small value of he E_int_^CP^ + ΔZPVE differences between both type complexes as the DFT (APFD, M062X, M062X + GD3, B3LYP, B3LYP + GD3) and MP2 method.

As the electrostatic energy is the largest contributor to the total interaction energy, the weakening of stabilization can be associated with a decreasing value of the dipole moment for hydrogen halides. Values of the dipole moment for XeOF_2_ and HF, HCl, HBr and HI calculated using M062X functional are 2.735D and 1.839, 1.113, 0.881, 0.467D, respectively.

### Infrared frequencies

Both computationally characterized structures depict a hydrogen-bonded complex, where the estimated H-X vibrational frequencies exhibit large shifts to lower wavenumbers (see Table 6). Shift magnitudes diminish going from smaller halogen atoms to the larger ones. This reflects decreasing electronegativity of the halogen atoms with the size increase. This is also reflected in calculated partial charges of the HX halogen atoms. The Mulliken charges for both structures calculated with the M062X functional are very similar: F: −0.37e (I), −0.38e (II), Cl: −0.33e (I), −0.31e (II), Br: −0.28e (I), −0.27e (II), −0.09e (I), −0.11e (II). Two types of complexes show different vibrational shifts of the Xe=O bond. In the type I, all HX molecules induce an upward vibrational shift, whereas in the type II the effect is the opposite (see Table 6). Such an effect can be caused by an interaction between the halogen atom of the HX moiety with the Xe atom, resulting in the strengthening of the Xe=O bond. Delocalized electron density between two complex subunits is observed, which also explains slightly smaller vibrational shifts when going from F to I, i.e. in the decreasing order of the halogen atom electronegativity.

**Table 6:** Vibrational stretching frequency shifts (in cm^−1^) for the ν(H-X), ν(Xe=O), ν_asym_(Xe-F) and ν_asym_(Xe-F) vibrations

vib:	ν(H-X)							
mol:	HF		HCl		HBr		HI	
Type: ^a^	I	II	I	II	I	II	I	II
MP2	−260	−430	−81	−222	−47	−202	−21	−136
APFD	−282	−355	−70	−288	−21	−273	−8	−174
M062X	−190	−331	−88	−213	14	−121	−89	−96
M062X*	−190	−332	−84	−211	15	−120	−88	−94
B3LYP	−277	−375	−62	−194	−7	−151	−1	−84
B3LYP*	−246	−360	−70	−197	−41	−173	−4	−86
B2PLYP	−268	−349	−72	−193	−25	−164	−12	−87
B2PLYP*	−247	−336	−76	−248	−92	−165	23	−51
vib:	ν(Xe=O)							
mol:	HF		HCl		HBr		HI	
Type:	I	II	I	II	I	II	I	II
MP2	12	−43	5	−30	4	−28	3	−20
APFD	13	−17	9	−17	8	−15	3	−11
M062X	15	−15	8	−8	5	−7	3	−4
M062X*	15	−15	7	−8	5	−7	3	−4
B3LYP	14	−19	10	−14	10	−12	2	−7
B3LYP*	14	−19	9	−12	10	−10	8	−5
B2PLYP	12	−29	7	−18	5	−17	1	−11
B2PLYP*	12	−28	8	−17	6	−15	3	−10
vib:	ν_asym_(Xe-F)							
mol:	HF		HCl		HBr		HI	
Type:	I	II	I	II	I	II	I	II
MP2	−99	−48	−290	−174	−306	−204	−349	−262
APFD	−93	−38	−300	−153	−296	−169	−332	−264
M062X	−97	−46	−300	−257	−159	−87	−272	−262
M062X*	−97	−45	−300	−257	−160	−88	−272	−262
B3LYP	−90	−42	−291	−202	−310	−230	−354	−302
B3LYP*	−90	−42	−286	−211	−307	−234	−327	−294
B2PLYP	−97	−48	−282	−203	−310	−229	−355	−296
B2PLYP*	−97	−49	−280	−209	−307	−234	−354	−293

^a^type I complex is stabilized by X-H^…^F hydrogen bond and X^…^Xe interaction; type II complex is stabilized by F-H^…^O

Estimated vibrational shifts of the Xe-F bonds in XeOF_2_ upon complexation are shown in Table 6. For both structures, the Xe-F vibrational modes display a downward shift as compared to the monomer values at all computational levels. The magnitudes of ν_asym_(Xe-F) vibrational shifts increase when going from F to I. All the calculated vibrational shifts indicate a hydrogen-bonded complex, in which an increased interaction between a positively charged hydrogen decides on the interaction direction and stretches the Xe-F bond via electron density delocalization to the space between the complex subunits. For the type I larger vibrational shifts are observed. Hydrogen bonded interaction is prevalent in the type I complexes, however the X^…^Xe interaction is also present. The latter does not appear in the type II complexes (according to AIM results), resulting in a deformation of the subunit structures.

All theoretically predicted vibrational shifts indicate the hydrogen-bonding interaction between the subunits as the main interaction channel, with existing interaction between a halogen atom of the HX moiety and the Xe atom of XeOF_2_. These features are also noticeable in the calculated structures of studied complexes, with the type II complexes more tilted from the HX halogen tail towards the XeOF_2_ subunit. Analysis of the F_2_OXe^…^HF electron density confirms the interaction patterns above, showing the bond critical points (BCP) in the space between xenon and the halogen atom of the HX subunit.

### Topological analysis of ρ(r), |RDG(r)| and η(r) fields

In the light of topological analysis of electron localization function, ELF, local electronic structures of the F_2_OXe^…^HX complexes, both types I and II, are represented by a set of core and valence attractors, constituting a sum of two attractor sets, localized separately for the XeOF_2_ and HX (X= F, Cl, Br, I) molecules. Since topologies of η(r) field are similar for different hydrogen halides, interacting with XeOF_2_, only complexes formed by the simplest HF molecules will be discussed in detail.

For the F_2_OXe^…^HF complex (type I) neither new disynaptic bonding, V(H,F) nor V(Xe,F) attractors are observed in the regions of H^…^F and Xe^…^F interactions. This shows that no new covalent bonds are formed upon the complex formation. Similarly, no bonding attractor is found in the region of H^…^O interaction in the type II complex.

**Fig. 3:**
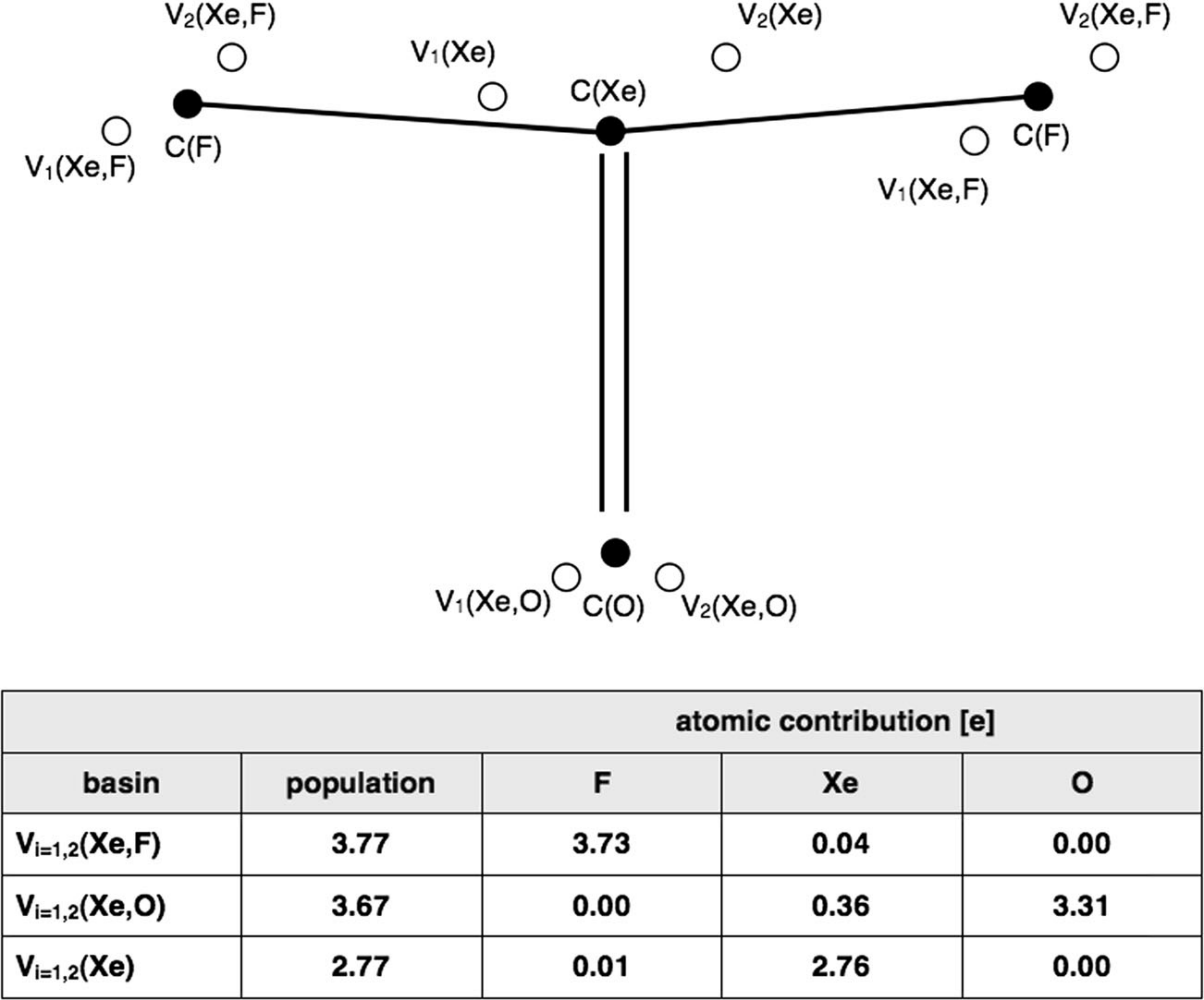
The core and valence attractors of the η(r) field together with the basin populations (in e) for the XeOF_2_ molecule. Note that all the valence attractors are localized below and above the symmetry plane and no bonding attractors are observed

Electronic structure of the isolated XeOF_2_ molecule is represented by four core attractors corresponding to oxygen, C(O), xenon, C(Xe) and fluorine, C(F) cores. In the valence space the disynaptic and monosynaptic non-bonding attractors are observed: two sets of V_i=1,2_(Xe,F), V_i=1,2_(Xe,O) and V_i=1,2_(Xe) localized below and above the molecular plane. All attractors and values of the basin populations () are shown in Fig. 3. The V_i=1,2_(Xe) attractors characterize the non-bonding electron density in the valence shell of Xe. These attractors can be associated with classical Lewis lone pairs. The localization of the disynaptic attractors in space, V_i=1,2_(Xe,F), V_i=1,2_(Xe,O), (far from bonding regions) suggests that those attractors characterize non-bonding electron density rather than the Xe-F and Xe=O chemical bonds. Combined analysis of the ρ(r) and η(r) fields, as proposed by Raub and Jansen [31], shows that the V_i_(Xe,F) and V_i_(Xe,O) basins contain electron density, coming exclusively from the fluorine (99 %) and oxygen (90 %) atoms. Atomic contributions to the V_i_(Xe,F) and V_i_(Xe,O) basins are shown in Fig. 3. Thus the V_i_(Xe,F) and V_i_(Xe,O) basins display the non-bonding characteristics of the V(F) and V(O) basins, respectively. It is worth emphasizing that the absence of shared electron density in the bonding basins, V_i_(Xe,F) and V_i_(Xe,O), unambiguously shows that the typical covalent Xe-F and Xe=O bonds as predicted by the Lewis formula, are not confirmed by topological analysis of ELF. It is evident that the electron densities of the xenon-fluorine and xenon-oxygen interactions are largely delocalized. Such characteristic suggests the charge-shift model of resonating electron density as a good explanation of its nature using the valence bond view of chemical bonding. Recent study on the bonding in the XeF_2_ molecule by Braïda and Hiberty [32] has shown that the charge-shift bonding, characterized by the dominant large covalent-ionic interaction energy, is a major stabilizing factor.

The AIM analysis carried out for the type I complex shows that BCP localized for the H^…^F interaction displays the largest ellipticity, ε_BCP,_ (0.310) for all the BCPs (see Table 3). Such high degree of electron density delocalization can be caused by close proximity of the (3,+1) CP (see Fig. 2). Total energy density, H_BCP_ is 0.003 hartree/bohr^3^, thus kinetic energy is a slightly dominant factor for the BCP, confirming a closed-shell interaction type, typical for hydrogen bonds. This conclusion is also supported by a very small average number of electron pairs delocalized (shared) between the F and H atoms (bond index, DI = 0.035). The non-covalent interaction, Xe^…^F, stabilizing the complex has similarly large value of ε_BCP_ (0.227). Such high value of electron density delocalization can also be explained by the proximity of the (3,+1) CP. Non-covalent character of interaction is shown by a small average number of electron pairs delocalized between Xe and F atoms (0.087). The type II F_2_OXe^…^HF complex is bound only by a single F-H^…^O hydrogen bond. The BCP characteristics for the H^…^O interaction are totally different from that observed for the H^…^F interaction (type I). Electron density delocalization for the BCP is much smaller - the value of the ε_BCP_ is 0.058 and the value of H_BCP_ is slightly negative (−0.003 hartree/bohr^3^). The non-covalent character of the interaction is associated with a relatively small average number of electron pairs, delocalized between the O and H atoms. It is 0.058, approximately half way between the value calculated for the H^…^F and Xe^…^F interactions.

**Fig. 4:**
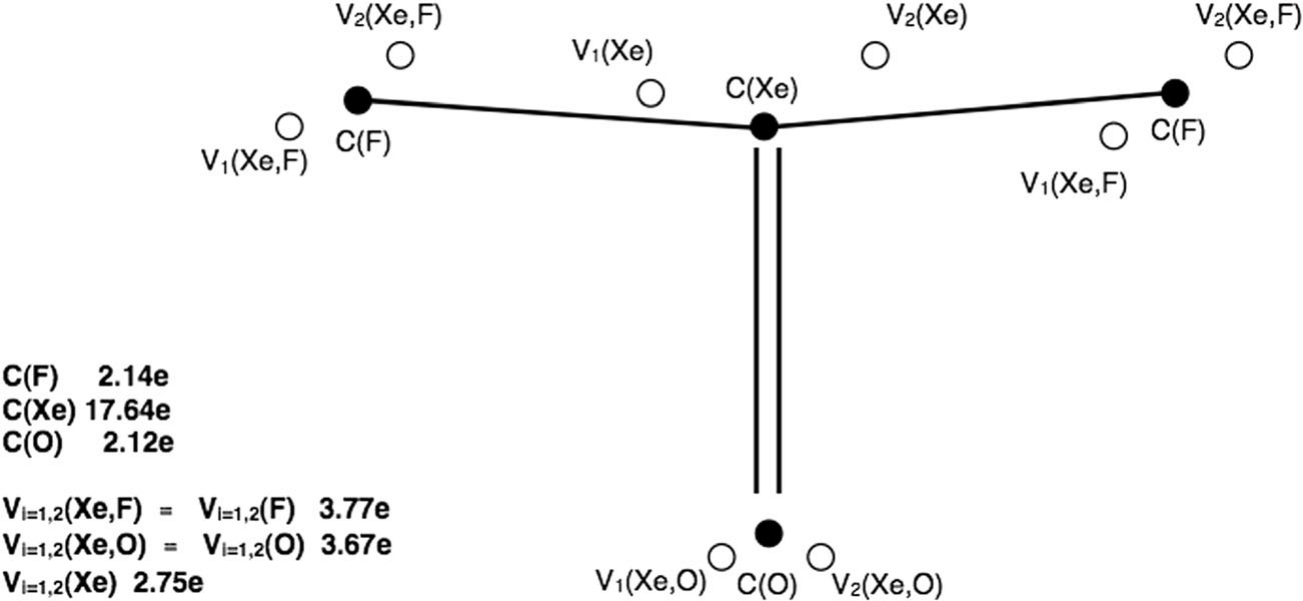
2D and relief maps of the reduced density gradients for the F_2_OXe^…^HF complexes. The bond paths of ρ(r) field are shown for the type II structure

In order to support our findings, we performed additional calculations using reduced density gradient. 2D plot and the relief map of reduced density gradient magnitude, |RDG(r)| for both structures are shown in Fig. 4. For the type I complex distinctive planar regions clearly exist and they are situated almost perpendicularly to the gradient paths of ρ(r) that join the attractor nuclei, F, H and Xe, F. Those regions are situated near the BCPs (ρ(r) field) characterizing the non-bonding H^…^F and Xe^…^F interactions. Thus both topological analysis of ρ(r) and topographical analysis of |RDG(r)| indicate the existence of both types of intermolecular interactions (I and II). A very similar picture has been obtained for the type II complex, with the planar region situated perpendicularly to the gradient path joining H and O nuclei attractors. However, this region also comprises interaction between the Xe and F atoms, where BCP of ρ(r) field is not observed. This suggests that the Xe^…^F interaction is also present in the type II complex, but is weaker than the H^…^F interaction. As reported by Contreras-Garcia et al. [33] sometimes there is no direct comparison between obtained BCPs of ρ(r) and the |RDG(r)| isosurfaces.

### The SAPT analysis

Nature of the non-covalent interactions in the F_2_OXe^…^HX complexes has been investigated using the symmetry-adapted intermolecular perturbation theory (SAPT). This approach calculates the total interaction energy between molecules as a sum of individual first and second order interactions with a clear physical interpretation. Selected components of total interaction energy are collected in Table 7. SAPT enables clear separation of electrostatic E_elst_^(1)^, induction E_ind_^(2)^ and dispersion E_disp_^(2)^ terms together with their respective exchange counterparts E_exch_^(1)^, E_ind-exch_^(2)^, E_disp-exch_^(2)^. The latter ones are sometimes denoted as Pauli repulsion due to electron exchange between monomers, when the molecules are close to each other. The SAPT0 and SAPT2 expressions discussed in this paper are defined as follows:

$$ {{\mathrm{E}}_{\mathrm{int}}}^{\mathrm{HF}} = {\mathrm{E}}_{\mathrm{comp}}\hbox{-} {\mathrm{E}}_{\mathrm{m}1}\hbox{-} {\mathrm{E}}_{\mathrm{m}2}{\updelta_{\mathrm{int}}}^{\mathrm{HF}}{{ = \mathrm{E}}_{\mathrm{int}}}^{\mathrm{HF}}{{\hbox{-}\ \mathrm{E}}_{\mathrm{elst}}}^{(1)}{{\hbox{-}\ \mathrm{E}}_{\mathrm{exch}}}^{(1)}{{\hbox{-}\ \mathrm{E}}_{\mathrm{ind}}}^{(2)}{{\hbox{-}\ \mathrm{E}}_{\mathrm{ind}\hbox{-} \mathrm{exch}}}^{(2)}{{\mathrm{E}}_{\mathrm{int}}}^{\mathrm{SAPT}0}{{ = \mathrm{E}}_{\mathrm{elst}}}^{(1)}{{ + \mathrm{E}}_{\mathrm{exch}}}^{(1)}{{ + \mathrm{E}}_{\mathrm{ind}}}^{(2)}{{ + \mathrm{E}}_{\mathrm{ind}\hbox{-} \mathrm{exch}}}^{(2)}{{ + \mathrm{E}}_{\mathrm{disp}}}^{(2)}{{ + \mathrm{E}}_{\mathrm{disp}\hbox{-} \mathrm{exch}}}^{(2)}{{\mathrm{E}}_{\mathrm{int}}}^{\mathrm{SAPT}2}{{ = \mathrm{E}}_{\mathrm{int}}}^{\mathrm{SAPT}0}{{ + \updelta}_{\mathrm{int}}}^{\mathrm{HF}}{{ = \mathrm{E}}_{\mathrm{int}}}^{\mathrm{HF}}{{ + \mathrm{E}}_{\mathrm{disp}}}^{(2)}{{ + \mathrm{E}}_{\mathrm{disp}\hbox{-} \mathrm{exch}}}^{(2)} $$

**Table 7:** Interaction energy components (in kcal/mol) calculated using SAPT for the F_2_OXe^…^HF complex. Calculations have been performed using the Def2-TZVPPD basis set

Component / structure	Type I	Type II
E_elst_^(1)^	−12.12	−9.95
E_exch_^(1)^	10.06	10.50
E_ind_^(2)^	−6.81	−6.68
E_disp_^(2)^	−3.66	−3.69
E_ind-exch_^(2)^	3.93	3.47
E_disp-exch_^(2)^	0.62	0.60
δ_int_^HF^	−0.59	−1.33
E_int_^HF^	−5.53	−3.99
E_int_^SAPT0^	−7.98	−5.75
E_int_^SAPT2^	−8.57	−7.08

For the F_2_OXe^…^HF complex, SAPT2 calculations using the Def2-TZVPPD basis set shows that the type I complex (F-H^…^F and Xe^…^F interactions) is more stable (−8.57 kcal/mol) than the type II complex (−7.08 kcal/mol). The interaction energies calculated at both SAPT0 and SAPT2 levels are similar to those obtained at the DFT and MP2 levels using the supramolecular approach (see Table 4). As can be seen, the δ_int_^HF^ terms for these complexes are not very high (less than 19 % of final E_int_^SAPT2^ values) for both type I and type II structures. The interaction energy decomposition results are similar for both types of F_2_OXe^…^HF complexes. The electrostatic term, E_elst_^(1)^ is the dominant stabilizing component for both structures. The values of E_elst_^(1)^ for both the type I structure (−12.12 kcal/mol) and the type II (−9.95 kcal/ mol) are larger than the total SAPT energy values for both of them (E_int_^SAPT0^, E_int_^SAPT2^). Introduction of the exchange contribution at the first SAPT order for the type I structure shows higher stabilization of the complex (E_elst_^(1)^ + E_exch_^(1)^ = −2.06 kcal/mol). For the type II complex, the exchange contribution is slightly (0.55 kcal/mol) bigger than the electrostatic energy. This confirms the stability of the complex formed with the F-H^…^F and Xe^…^F interactions (type I), even without including the electron correlation correction. For the type II complex, stabilized only by the F-H^…^O hydrogen bond, the electron correlation needs to be included in order to obtain a reliable picture.

The electric polarization caused by nuclear and electron cloud charges largely influence intermolecular interactions. Thus, the induction energy, E_ind_^(2)^, is the biggest contributor to the total SAPT energy at the second-order for both complexes (type I and type II). It is, however, still smaller than the electrostatic effect. The E_ind-exch_^(2)^ contribution is a compensation to the E_ind_^(2)^ term, whereas the E_ind-exch_^(2)^ values are roughly half of the E_ind_^(2)^ absolute value for both complexes. Even if the differences between the E_ind_^(2)^ absolute values for the type I and type II complexes are negligible (0.07 kcal/mol), the total SAPT2 energy (E_int_^SAPT2^) for the type I complex is lower than for the type II. Thus E_ind_^(2)^ contributes less to the type I E_int_^SAPT2^ than for the type II. Absolute values of the dispersion energy, E_disp_^(2)^ for the type I and type II complexes - the attractive energy determined by mutual interactions of the induced multiple moments in both molecules - are almost equal, −3.66 and −3.69 kcal/mol, respectively. Contribution of the dispersion energy to the E_int_^SAPT2^ energy is about 43 % for the type I and 52 % for type II. The E_disp-exch_^(2)^ term, the compensation term to the E_disp_^(2)^, has quite significant influence on the total interaction energy, compensating E_disp_^(2)^ by about 17 % (type I) and 16 % (type II). The E_ind_^(2)^/ E_disp_^(2)^ ratio is an effective measure of a relationship between induction and dispersion effects. Calculated ratios of E_ind_^(2)^/ E_disp_^(2)^ for the type I and type II F_2_OXe^…^HF complex are 1.86 and 1.81, respectively. The type I complex is therefore more favorable than the type II complex.

## Conclusions

The nature of chemical bonds and intermolecular interactions formed by noble gases deserve special attention, due to group 18 relative unreactivity. New compounds and intermolecular complexes are being constantly researched for. Identification of the F_2_OXe^…^HF complex by Schrobilgen’s group [3] constitutes a very interesting example in the area. This paper presents a detailed description of geometrical structures, energetics and infrared spectra of the intermolecular complexes of XeOF_2_ with hydrogen halides, F_2_OXe^…^HX (X= F, Cl, Br, I). Our research shows that combined application of the quantum chemical topology methods, namely topological analysis of electron density, reduced density gradient and electron localization function (in real space) provide a complete description of the electronic structure of the F_2_OXe^…^HF complex. Topological studies have been complemented with the interaction energy decomposition analysis (SAPT), based on the molecular orbitals in the Hilbert space. Not only such an approach does offer a deeper insight into the nature of chemical bonds and weak interactions (H^…^F, H^…^O, Xe^…^F), playing key role in the F_2_OXe^…^HF stability, but takes into account the components with physical meaning as well.

We would like to summarize our results as follows:

1. Geometrical structure optimizations for the F_2_OXe^…^HX (X= F, Cl, Br, I) complexes yield two minima on the PES. One, where the hydrogen halide is bound to XeOF_2_ by X-H^…^F hydrogen bond and Xe^…^X interaction (type I), and another where the X-H^…^O hydrogen bond (type II) mainly stabilizes the structure.
2. The interaction between the XeOF_2_ and HX molecules in the F_2_OXe^…^HX (X= F, Cl, Br, I) complexes is strongly dependent on the electron density functional chosen and the type of halogen atom. The relative stability (ΔE_int_^CP^ + ΔZPVE) between the type I and II complexes lies within a range of less than 1 kcal/mol (except for F_2_OXe^…^HF, B2PLYP, B2PLYP + GD3).
3. Existence of the H^…^F, Xe^…^F (type I) and H^…^O (type II) intermolecular interactions stabilizing the F_2_OXe^…^HF complex is indicated by respective critical points of index 1 (BCPs) and atomic interaction lines in the field of ρ(r). All interactions are of closed-shell type (ρ_BCP_(r) < 0.04 e/bohr^3^, ∇^2^ρ_BCP_(r) > 0, |∇^2^ρ_BCP_(r)| < 0.13 e/bohr^5^, δ(A,B) < 0.09).
4. Topological analysis of ELF performed for the isolated XeOF_2_ molecule using DFT method does not show any bonding attractors or basins for formally single (Xe-F) and double (Xe=O) bonds, thus typical covalent bonds based on sharing electron density are not present. The maxima of ELF (attractors) in valence space are localized only in regions, where formal lone pairs (non-bonding electron densities) are expected.
5. From the ELF perspective electron densities in the xenon-fluorine and xenon-oxygen bonds are largely delocalized. Both bonds can possibly be classified as charge-shift bonds. Furthermore, values of ELF for the BCPs, localized in the regions of the Xe-F and Xe=O bonds, are relatively large for the (3,-1) CP (η(r) ≈ 0.2 for Xe-F, η(r) ≈ 0.6 for Xe=O, M062X). Therefore they cannot be described as typical ionic bonds.
6. The first order SAPT analysis shows that the value of the interaction energy (E_elst_^(1)^ + E_exch_^(1)^) for the type I complex is negative, but slightly positive for the type II complex. Thus for the complex stabilized only by the X-H^…^O hydrogen bond, electron correlation correction is essential in order to obtain reliable energy results.
7. The second order of SAPT analysis shows that the induction energy term, E_ind_^(2)^, is the biggest contribution to total SAPT energy, thus the electric polarization caused by both electron cloud and the nuclear charges have significant influence on the intermolecular interactions.
